# Localized comedones and perifollicular hyperpigmentation during tapinarof cream therapy: a case series

**DOI:** 10.1093/skinhd/vzag121

**Published:** 2026-07-31

**Authors:** Yusuke Sano, Yasuaki Ogura, Hiroki Morimoto, Naoko Funai, Tetsuya Honda

**Affiliations:** Department of Dermatology, Hamamatsu University School of Medicine, Hamamatsu, Japan; Department of Dermatology, Hamamatsu University School of Medicine, Hamamatsu, Japan; Department of Dermatology, Hamamatsu University School of Medicine, Hamamatsu, Japan; Division of Dermatology, Hamamatsu Rosai Hospital, Hamamatsu, Japan; Department of Dermatology, Hamamatsu University School of Medicine, Hamamatsu, Japan

## Abstract

Tapinarof is an aryl hydrocarbon receptor agonist approved for the treatment of atopic dermatitis (AD) and psoriasis. While common adverse events include headache and folliculitis, comedones and perifollicular hyperpigmentation are under-recognized. Here, we report eight patients with AD or psoriasis who developed comedones and/or perifollicular hyperpigmentation during therapy. Lesions developed primarily at application sites; however, in one patient, lesions occurred at a distant site. Lesions appeared on the face in six patients, the legs in one and the arms in one. Time of onset ranged from 1 to 8 months after initiation of tapinarof therapy. Tapinarof was discontinued in all cases. Three patients were treated with topical adapalene/benzoyl peroxide, with partial improvement in one and minimal improvement in two. One patient showed gradual improvement without additional treatment, whereas the remaining five showed no or minimal improvement during follow-up. Our cases provide important information on under-recognized adverse events during tapinarof therapy.

## Teaser

We report a case series of eight patients with atopic dermatitis or psoriasis who developed localized comedones and/or perifollicular hyperpigmentation during tapinarof cream therapy, predominantly at application sites but also, in some cases, at distant nonapplication areas. The lesions most frequently involved the facial region, particularly the periorbital area, and in several patients persisted with minimal improvement after discontinuation of treatment. These findings highlight underrecognized follicular adverse reactions to tapinarof and underscore the need for careful monitoring, especially when used on facial skin.

What is already known about this topic?

- Tapinarof therapy is associated with follicular adverse events, including acneiform eruptions and folliculitis.
- However, the detailed clinical features and outcomes of other follicular reactions remain poorly characterized.

What does this study add?

- We describe comedones and/or perifollicular hyperpigmentation developing during tapinarof cream therapy, sometimes at sites distant from application.
- These reactions predominantly involved the facial, especially periorbital, area and often showed slow or incomplete resolution after discontinuation.

Tapinarof is a newly approved topical aryl hydrocarbon receptor (AhR) modulator for atopic dermatitis (AD) and psoriasis.^1,2^ Through AhR activation, tapinarof induces antioxidative and anti-inflammatory pathways, at least in part through interactions with Nrf2- and nuclear factor-κB-related signalling, resulting in significant improvement in AD and psoriasis.^3^ Importantly, AhR is a ligand-dependent transcription factor whose downstream effects vary depending on the ligand and cellular context. In the skin, AhR activation by ligands, including tapinarof, coal tar and FICZ [6-formylindolo(3,2-b)carbazole], has been associated with epidermal differentiation, follicular keratinization and melanogenesis.^4^

Headache and folliculitis are common adverse events of tapinarof therapy.^3^ Other adverse events, including follicular keratinization and hyperpigmentation, have been reported in clinical trials;^1,2^ however, their clinical characteristics and outcomes have not been well described.

Here, we report eight patients with AD or psoriasis who developed comedones and perifollicular hyperpigmentation during tapinarof therapy, highlighting under-recognized cutaneous reactions that require careful monitoring.

## Case report

### Case 1

A 52-year-old man with AD initiated tapinarof for facial erythema during dupilumab therapy (Figure 1a). Dupilumab was subsequently discontinued. After 2 months, erythema improved; however, multiple noninflammatory comedones with surrounding hyperpigmentation appeared in the left periocular area (Figure 1b). Tapinarof was discontinued. Two months after discontinuation, the comedones and hyperpigmentation gradually improved (Figure 1c), and although further improvement was observed at 4 months, milia appeared (Figure 1d).

**Figure 1:**
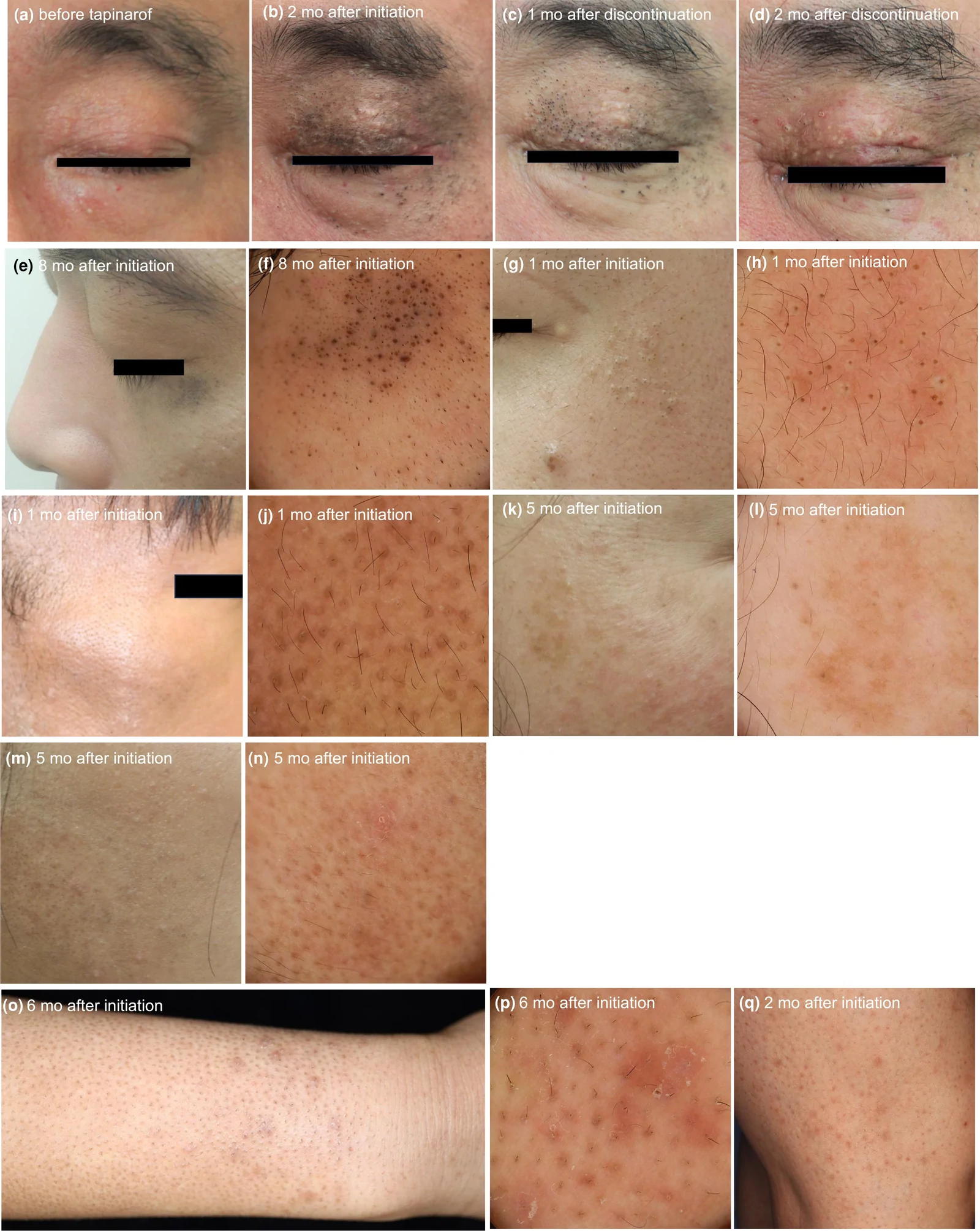
Clinical and dermoscopic findings of tapinarof-associated follicular reactions. (a–d) Case 1: clinical appearance before tapinarof therapy (a) and 2 months after initiation (b). Multiple periocular comedones and pigmentation developed in the left periocular area (b). One month (c) and 2 months (d) after discontinuation of tapinarof therapy. (e, f) Case 2: 8 months after the initiation of tapinarof therapy, multiple comedones developed in the left lower periocular area (e). Dermoscopic findings of the comedones (f). (g, h) Case 3: comedones on the cheek (g). Dermoscopy reveals multiple comedones (h). (i, j) Case 4: perifollicular hyperpigmentation on the temples (i). Dermoscopy confirms localized perifollicular hyperpigmentation (j). (k, l) Case 5: periocular comedones at a site distant from the tapinarof application area (k). Dermoscopy reveals perifollicular hyperpigmentation (l). (m, n) Case 6: noninflammatory closed comedones on the cheek (m). Dermoscopy reveals perifollicular hyperpigmentation (n). (o, p) Case 7: perifollicular hyperpigmentation on the forearm (o). Dermoscopy reveals localized follicular keratinization and pigmentation (p). (q) Case 8: perifollicular hyperpigmentation with folliculitis-like papules on the lower leg. mo, months.

### Case 2

A 34-year-old man with AD initiated dupilumab with tapinarof for facial lesions. Eight months later, noninflammatory comedones appeared on both lower eyelids (Figure 1e, f). Tapinarof was discontinued and topical benzoyl peroxide was initiated. The lesion persisted but partially improved 2 months later.

### Case 3

A 34-year-old man with AD, managed with topical therapies for many years, applied tapinarof to facial lesions. One month later, noninflammatory comedones appeared on both lower eyelids (Figure 1g, h). Tapinarof was discontinued, topical benzoyl peroxide was initiated and partial improvement was observed after 6 months.

### Case 4

A 45-year-old man with AD had received topical corticosteroid therapy for the facial lesions. After lesion resolution, therapy was switched to tapinarof. One month later, perifollicular hyperpigmentation developed at the temples. Tapinarof was discontinued, and treatment was switched to tacrolimus (Figure 1i, j).

### Case 5

A 42-year-old woman with AD and facial vitiligo involving the forehead initiated tapinarof after inadequate response to topical corticosteroids, tacrolimus and delgocitinib. Five months later, noninflammatory comedones developed in the periocular area, where tapinarof had not been applied. Tapinarof was discontinued (Figure 1k, l), and topical adapalene/benzoyl peroxide was initiated. Minimal improvement was observed after 2 months, with slight improvement noted after 4 months.

### Case 6

A 41-year-old woman with AD initiated tapinarof for facial lesions after discontinuation of tacrolimus due to irritation. Four months later, noninflammatory closed comedones and perifollicular hyperpigmentation appeared on the cheek (Figure 1m, n). Tapinarof was discontinued, and topical adapalene/benzoyl peroxide was initiated; however, minimal improvement was observed after 2 months.

### Case 7

A 38-year-old woman with AD receiving dupilumab for 5 years initiated tapinarof for arm lesions. Six months later, perifollicular hyperpigmentation was observed at the application sites (Figure 1o, p). Tapinarof was discontinued, and the patient was managed with observational follow-up. Minimal improvement was observed after 2 months.

### Case 8

A 41-year-old woman with psoriasis and psoriatic arthritis, treated with adalimumab for 4 years after inadequate response to apremilast, initiated tapinarof for residual psoriatic lesions. Two months later, perifollicular hyperpigmentation and folliculitis-like papules developed at the application sites (Figure 1q). Tapinarof was discontinued, topical nadifloxacin was initiated and the lesions gradually improved over 6 months.

Patient demographic and clinical characteristics are summarized in Table 1. No patients had active acne, recurrent acne requiring treatment or similar follicular lesions before tapinarof initiation

**Table 1:** Patient demographics and clinical characteristics

Case	Age (years)/sex	Disease	Lesion location	Duration of tapinarof therapy before onset of AE (months)	AE	Management	Follow-up duration after discontinuation (months)	Clinical course	Naranjo ADR probability scale
1	52/M	AD	Periocular	2	Noninflammatory comedones; hyperpigmentation	Observation	4	Improved, with subsequent milia formation	5
2	34/M	AD	Lower eyelids	8	Noninflammatory comedones	Topical benzoyl peroxide	2	Partial improvement	5
3	34/M	AD	Lower eyelids	1	Noninflammatory comedones	Topical benzoyl peroxide	6	Partial improvement	5
4	45/M	AD	Temples	1	Perifollicular hyperpigmentation	Topical tacrolimus	2	No or minimal improvement	4
5	42/F	AD, vitiligo	Periocular^a^	5	Noninflammatory comedones	Topical adapalene/benzoyl peroxide	4	Partial improvement	3
6	41/F	AD	Cheeks	4	Noninflammatory closed comedones; perifollicular hyperpigmentation	Topical adapalene/benzoyl peroxide	2	No or minimal improvement	4
7	38/F	AD	Arms	6	Perifollicular hyperpigmentation	Observation	2	No or minimal improvement	4
8	41/F	Psoriasis	Legs	2	Perifollicular hyperpigmentation; folliculitis-like papules	Topical nadifloxacin	6	Partial improvement	5

AD, atopic dermatitis; ADR, adverse drug reaction; AE, adverse event; F, female; M, male. ^a^Developed at a site distant from the tapinarof application area (forehead).

## Data Availability

The data will be shared on reasonable request to the corresponding author.

## Discussion

Clinical trials of tapinarof cream reported follicular events in 8.9–10.0% of patients with AD and 17.8–23.5% of those with psoriasis, with low discontinuation rates.^1,2^ In Japanese phase III trials, the discontinuation rate due to follicular reactions was approximately 1.0%.^5^ Although real-world safety data remain limited, a recent observational study reported folliculitis in 2.78% of patients treated with tapinarof monotherapy.^6^ However, these events were generally described using broader terms such as ‘folliculitis’ or ‘acne’, and detailed morphologies such as comedones and perifollicular hyperpigmentation were not well characterized. At our institution, among 209 patients prescribed tapinarof during the study period, 8 developed comedones and/or perifollicular hyperpigmentation, corresponding to an observed frequency of 3.8%.

Some patients were using or had previously used other medications, including dupilumab or topical corticosteroids. Although we found no evidence linking dupilumab to localized comedones or perifollicular hyperpigmentation similar to those in the present cases, topical corticosteroids can be associated with acneiform eruptions or pigmentary changes, particularly with prolonged or inappropriate use. Thus, the influence of concomitant or prior medications cannot be completely excluded. Nevertheless, because the lesions developed after tapinarof initiation and were located mainly at application sites in most cases, tapinarof was considered the most likely contributing factor.

In patients presenting with comedones, the lesions were clinically noninflammatory. Drug-induced acneiform eruptions have been reported with various topical and systemic agents,^7^ and altered follicular keratinization is considered one possible mechanism. However, the localized noninflammatory comedones and perifollicular hyperpigmentation observed here appear distinct from typical drug-induced acneiform eruptions, which are often characterized by inflammatory papules or papulopustules.^7^

Comedones and/or perifollicular hyperpigmentation were observed mainly on the face. Two patients showed perifollicular hyperpigmentation at nonfacial sites, whereas comedones were observed exclusively on the face, particularly in the periorbital area. Although tapinarof cream was applied to the entire face in several patients, comedones developed predominantly in the periorbital area. In one patient, lesions developed in the periorbital area, despite tapinarof being applied only to the forehead. These findings suggest that the periorbital area may be particularly susceptible to comedone development during tapinarof therapy.

The reason for this site-specific predilection remains unclear. The thin and structurally complex periorbital skin may facilitate penetration or local retention of topical agents.^8^ Regional differences in follicular structure, sebaceous activity and barrier ­properties may influence follicular occlusion, whereas repeated ­movement or rubbing may affect irritation or drug distribution. Ultraviolet light-related responses may contribute to facial pigmentation. Although regional differences in AhR responsiveness of follicular keratinocytes or melanocytes are possible, direct evidence in the periorbital area is lacking. Therefore, these interpretations should be made with caution.

The onset of comedones and perifollicular hyperpigmentation ranged from 1 to 8 months after the initiation of tapinarof therapy. Because these changes may be subtle, the actual onset may have preceded clinical recognition in some cases. After discontinuation, improvement was often slow or incomplete. These findings suggest that clinicians should inform patients about these potential adverse events and monitor carefully for their development and persistence.

Histological evaluation was not performed in our cases because consent for biopsy could not be obtained from the patients. Therefore, we could not confirm the precise follicular changes or directly assess AhR involvement, which substantially limits definitive interpretation of the underlying pathology. Future histopathological and mechanistic studies are required to clarify the underlying pathophysiology and long-term course of these lesions.

Collectively, these cases highlight previously under-recognized follicular adverse effects associated with tapinarof therapy. Clinicians should recognize comedones and perifollicular hyperpigmentation as tapinarof-associated follicular events, not only at application sites, but also at distant sites.
